# Assessment of quality of commercial hand sanitizers using Fourier transform infrared spectroscopy and gas chromatography

**DOI:** 10.1016/j.mex.2023.102274

**Published:** 2023-07-06

**Authors:** Saima Alam, Md. Masudur Rahman Rahat, Nusrat Jahan Upoma, Chandan Halder, Shyama Prosad Moulick, Md. Monarul Islam, Wenben Liu, Ahsan Habib

**Affiliations:** a Department of Chemistry, University of Dhaka, Dhaka 1000, Bangladesh; b Chemical Research Division, BCSIR Laboratories Dhaka, Bangladesh Council of Scientific and Industrial Research (BCSIR) Dhanmondi, Dhaka 1205, Bangladesh; c Research Center for Eco-Environmental Sciences, Chinese Academy of Sciences, Beijing 100085, China

**Keywords:** Fourier transform infrared and gas chromatography analysis of ethanol, isopropanol and methanol, COVID-19 pandemic, Hand sanitizers, Alcohol content, Infrared spectroscopy/gas chromatography, Quick and easy analysis

## Abstract

Since the beginning of the COVID-19 pandemic, the use and manufacture of alcohol-based hand sanitizers increased exponentially. Efficacy of hand sanitizers mainly depends on active ingredients like ethanol and isopropanol (IPA). Even though methanol is extremely hazardous to people, it is still illegally used in hand sanitizers in Bangladesh. Developing a quick and simple analytical method for detecting and quantifying ethanol/IPA/methanol is crucial. Here, Fourier transform infrared spectroscopy (FTIR) was used to identify and quantify alcohol content in commercially available hand sanitizers in a quick and easy way. Comparing the FTIR and GC data, provided quite similar results. Unlike previous studies by FTIR, C–H, CH_3_–C–CH_3_ stretching, and C–H bending vibrational modes were employed to construct analytical calibration curves to detect and quantify alcohol in hand sanitizers. According to FTIR and GC findings, ethanol and IPA content were found to be 43–82% and 40–69%, and 56–64% and 61–66%, respectively, whereas ethanol was labeled at 66–80% and IPA at 65–70%. FTIR and GC revealed methanol content ranging from 37 to 98 and 19 to 81%, respectively. Also, the FTIR was significantly faster than the GC. Therefore, FTIR can be used to commercially analyze the quality of hand sanitizers.

• FTIR was used to identify and quantify alcohol content in commercially available hand sanitizers in a quick and easy way.
• Comparing the FTIR and GC data, provided quite similar results.
• Out of ten samples, five contained ethanol, three IPA, and two methanol.

## Graphical abstract

**Figure:**
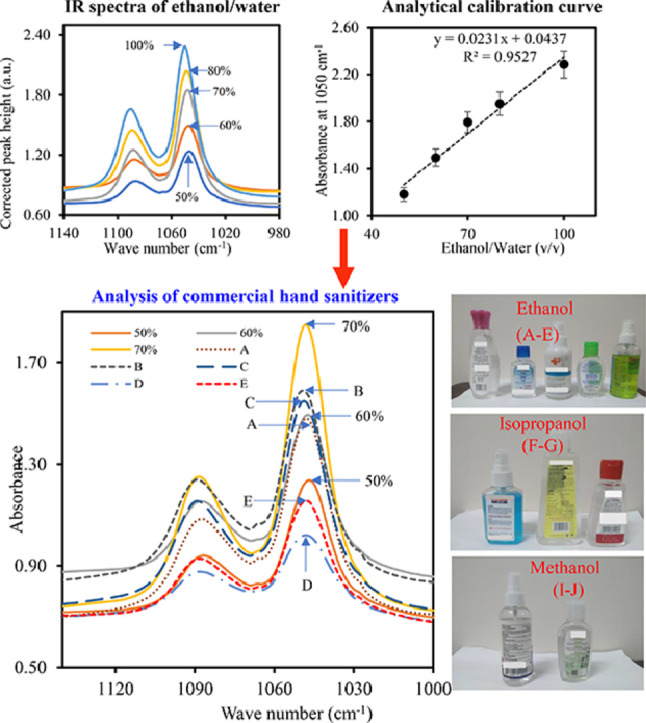

Specifications Table

**Table:** 

Subject area	Chemistry
More specific subject area	Analytical chemistry
Name of your method	Fourier transform infrared and gas chromatography analysis of ethanol, isopropanol and methanol
Name and reference of original method	Not applicable
Resource availability	Reagents: Ethanol, isopropanol (IPA), methanol, acetone 1-propanol and ethyl acetate were purchased from Active Fine Chemicals Ltd., Bangladesh. Commercially available hand sanitizers were purchased from local drug stores in Dhaka, Bangladesh. Methods: Fourier transform infrared spectrophotometer (FTIR) (IRPrestige-21, SHIMADZU, Japan) Gas chromatography-flame ionization detector (GC-FID) system (SCION-5 column, Stationary Phase -5%-phenyl-methylpolysiloxane; Internal diameter - (30 m x 0.25 mm) and film thickness - 0.25 µm)

## Method details

### Background

Due to COVID-19 pandemic, the World Health Organization (WHO) has encouraged the public to wash their hands regularly to avoid infection, prompting alcohol-based hand sanitizers to become increasingly popular. Therefore, these products were in short supply. To counteract this, regulatory agencies around the world, including the US Food and Drug Administration (FDA), European Union, and the Bangladesh Health Administration, issued guidelines for drug companies and pharmaceuticals to temporarily prepare these sanitizers in order to increase supply during the public health emergency. According to WHO recommendations, the formulation of hand sanitizers are as follows: formulation I: ethanol 80% v/v, glycerol 1.45% v/v, hydrogen peroxide 0.125% v/v and formulation II: isopropyl alcohol 75% v/v, glycerol 1.45% v/v, hydrogen peroxide 0.125% v/v [1], [2], [3], [4], [5]. This is due to the likelihood that alcohols damage microbial membranes and prevent metabolism through crucial processes including protein denaturation and lipid membrane disintegration [6]. During the COVID-19 pandemic, the concentration of active ingredients like ethanol and/or isopropanol did not fulfill WHO standards. Moreover, several hand sanitizers included methanol, which is toxic to humans. Consequently, several techniques, in particular gas chromatography/gas chromatography-mass spectrometry (GC/GC–MS) [7], [8], [9], [10], [11], [12], [13], [14], [15], [16], FTIR and Raman spectroscopy [17], [18], [19], [20], [21], [22], [23], were tested in order to develop a quick and accurate method for detecting and quantifying active ingredients in commercial hand sanitizers, such as ethanol or isopropanol and adulterate methanol.

The goal of this work was to develop an infrared spectroscopy-based analytical method for detecting and quantifying ethanol, isopropanol (IPA) or unwanted methanol in commercial hand sanitizers. The results obtained by the IR method were also compared using the gas chromatography method. In developing countries like Bangladesh, where the IR technique is available at universities, pharmaceutical companies, and law enforcement agencies, the IR-based developed method can be used as an indispensable analytical tool for analyzing hand sanitizers. Because IR spectroscopy is easier to use than GC and/or GC–MS, a miniature IR-based method for real-world applications can be developed.

### Chemicals and reagents

Ethanol, IPA, methanol and acetone were purchased from Active Fine Chemicals Ltd., Bangladesh. Commercially available hand sanitizers were purchased from local drug stores in Dhaka, Bangladesh in order to detect and quantify alcohol content (see Figure S1). Distilled water was used throughout the experiment.

### Methods

Calcium fluoride crystal plates were used as sample holder for liquid samples in infrared (IR) spectroscopy. A sample holder made of calcium fluoride crystals is shown in Figure S2. Calibration standards were prepared by diluting each absolute alcohol with water in the following proportions: 100/0, 80/20, 70/30, 60/40, and 50/50. Fourier transform infrared spectrophotometer (FTIR) (IRPrestige-21, SHIMADZU, Japan) was used to record the IR spectra of the calibration standards and the commercial hand sanitizers within a window range of 4000 to 400 cm^−1^ prior to analysis of the real sanitizer samples. About 20–30 μL of the standard was directly introduced into a capillary of the sample holder and then placed on the sample window of the FTIR instrument and scanned to record the IR spectrum. Each standard was scanned at least three times (n = 3).

### Analysis of commercial hand sanitizers

In a similar way, about 20–30 μL of the relevant sanitizer sample was directly loaded into a capillary of the sample holder and placed on the sample window of the FTIR instrument and then scanned. The raw data were transferred to a desktop and edited using Excel in order to re-generate the relevant spectrum. Similarly, the IR spectrum for each sample was scanned at least three times (n = 3). The average peak heights were obtained from the spectra and used for determining the alcohol content.

### GC-FID analysis of the samples

The content of ethanol, IPA and methanol were determined using a gas chromatography-flame ionization detector (GC-FID) system. Separation was achieved by GC column SCION-5 (5%-phenyl-methylpolysiloxane, internal diameter 30 m × 0.25 mm and film thickness 0.25 µm) at a flow rate of 1.5 mL/min.

**Fig. 1:**
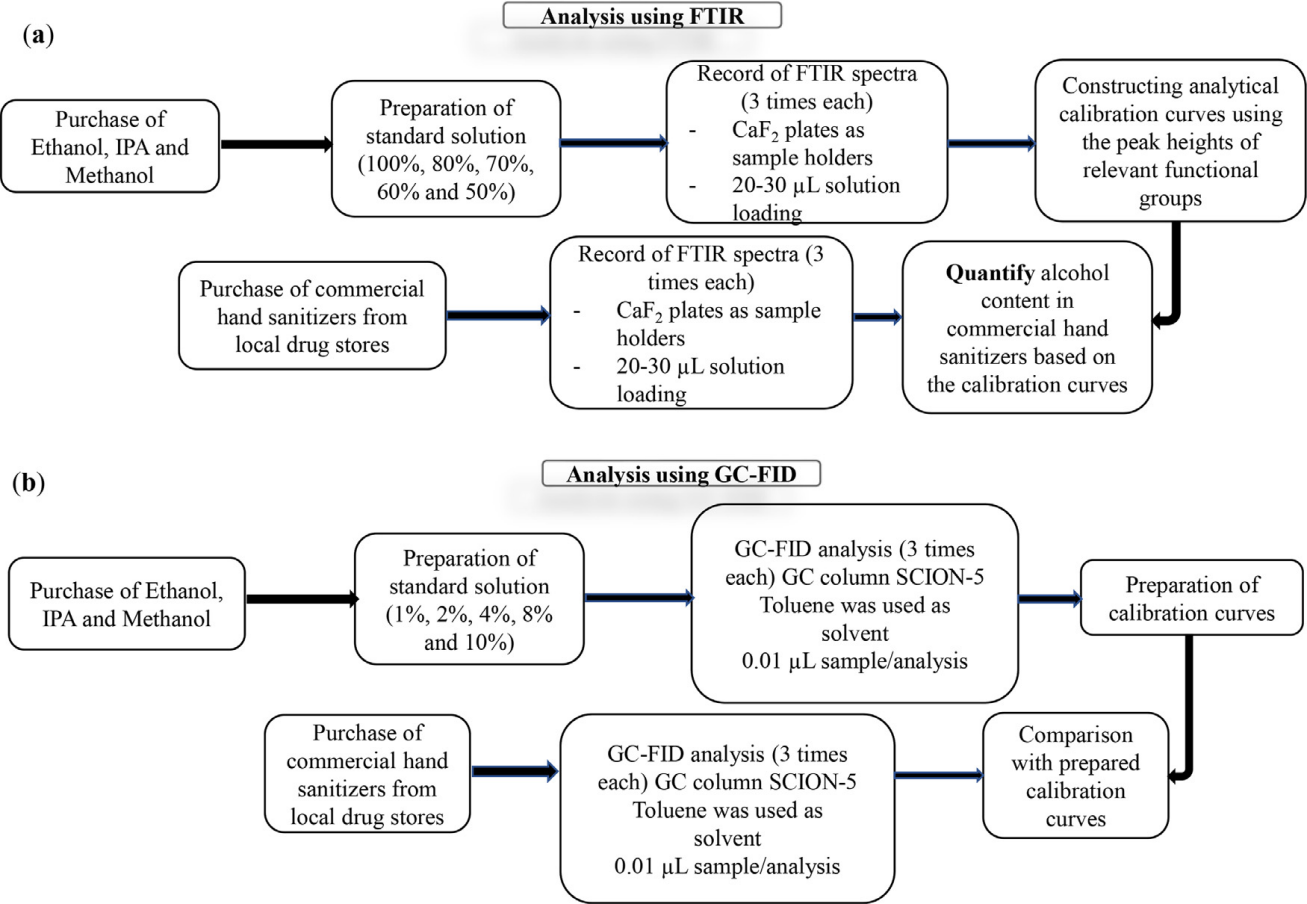
A scheme of experimental protocol for sample preparation and analysis using (a) FTIR and (b) GC-FID techniques, respectively.

To construct calibration curves, working standard solutions of ethanol, IPA, and methanol at various concentrations were prepared in toluene. Each of the hand sanitizer samples was diluted with toluene 25 times. Exactly, 0.01µL sample/standard was injected by auto injector to the column. All of these analyses were carried out in triplicate (n = 3) to ensure that the experiment was of high quality. Table S1 provides more information. Fig. 1 shows a scheme of experimental protocol for sample preparation and analysis using (a) FTIR and (b) GC-FID techniques, respectively.

### Analytical method validation

Alcohol content, standard deviation (SD), relative standard deviation (RSD) and linear range correlation coefficient (*R*^2^) were measured to validate the IR spectroscopy as an analytical method [24]. For each standard curve, at least three peak heights (n = 3) were used, and the generated curve was taken into account if the square correlation coefficients (*R*^2^) were greater than 0.905. The calibration curves were constructed using the average peak heights of the relevant alcohol's vibrational modes. Table 1 lists the pertinent modes of vibration, their wave numbers and the linear range correlation coefficient (*R*^2^) for each alcohol.

**Table 1:** Construction of analytical calibration curves. .

Alcohol	Modes of vibration	Wave number (cm^−1^)	$R^{2}$
Ethanol	C−H stretching	2976	0.9527
	C−H bending	881	0.9801
	C−O stretching	1050	0.9051
IPA	C−H stretching	2970	0.9129
	$CH_{3}-C-CH_{3}$ bending	952	0.9222
Methanol	C−H stretching	2943	0.9039
	C−O stretching	1032	0.9963

The relevant analytical calibration curves are shown in Figure S3 (C−O stretching, 1050 cm^−1^, for ethanol), Figures S4(a) and (b) (C−H stretching, 2976 cm^−1^, and C−H bending, 881 cm^−1^ for ethanol), Figures S5(a) and (b) (C−H stretching, 2970 cm^−1^, and CH_3_−C−CH_3_, 952 cm^−1^, for IPA) and Figures S6(a) and (b) (C−H stretching, 2943 cm^−1^, and C−O stretching, 1032 cm^−1^, for methanol).

Figures S7(a), (b) and (c) show the IR spectra of pure ethanol, IPA and methanol, respectively. As seen from Figures S7(a), (b) and (c), red and blue circles indicate C−H and C−O stretching and the purple circle shows C-H bending while green and orange circles represent −C(CH_3_)_2_ bending and C−C−O symmetric stretching, respectively [18]. In this study, these functional groups were considered in order to detect and quantify the different alcohols in alcohol-based commercial hand sanitizers. The C−O stretching vibrational mode, which appears between 1075 and 1350 cm^−1^, has been identified as a promising group for quantifying ethanol, isopropanol, and even methanol in commercial hand sanitizers [18,25]. Hydrogen bonding in alcoholic compounds plays vital role for the existence of alcohols as liquid. Water molecules in alcohols also forms hydrogen bonding with the alcoholic molecules. So, the presence of water alters the C−O group's intensity as well as its peak position. This is why the C−O stretching has been used as a key parameter in the construction of analytical calibration curves [25].

Water, on the other hand, has no direct interaction with the non-polar C−H moiety in alcohol, but it does affect the intensity of the C−H groups. In order to identify and quantify ethanol, isopropanol, and methanol in hand sanitizers, the C−O and C−H stretching and C−H bending vibrational modes, as well as the CH_3_−C−CH_3_ bending mode for isopropanol, were taken into account in the construction of analytical calibration curves.

**Fig. 2:**
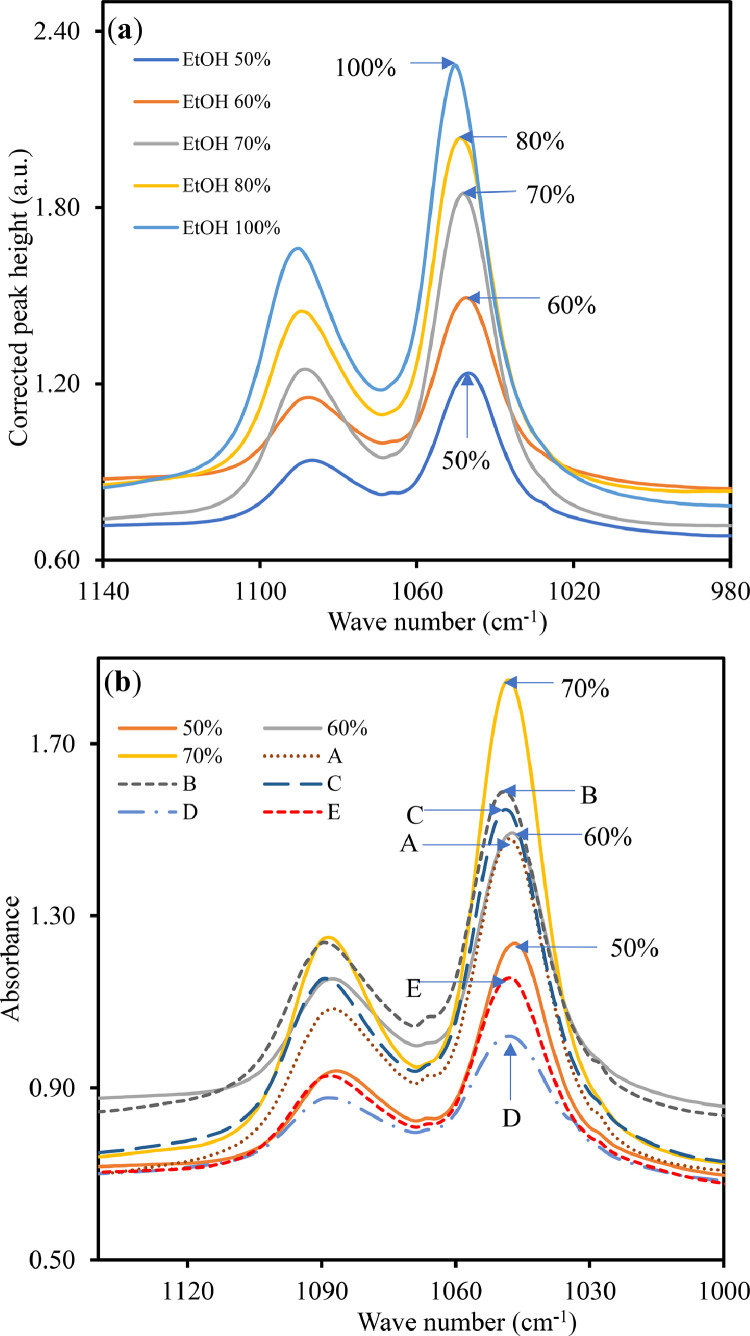
IR spectra of (a) ethanol standards and (b) commercial brands A, B, C, D, E and F with ethanol standards 50, 60 and 70%.

Figs. 2(a) and (b) show the IR spectra of ethanol standards and commercial brands A, B, C, D and E with ethanol standards of 50, 60, and 70%, from 980 to 1140 cm^−1^, respectively. The C−O group's absorbances centered at about 1050 cm^−1^ for various ethanol/water ratios were used to construct an analytical curve, as illustrated in Figure S7. The calibration curve had a moderately good correlation coefficient (*R*^2^) of 0.953 (Figure S3). As indicated in Fig. 2(b), the ethanol content of brands A, B, C, D, and E are 59, 63, 62, 35, and 45%, respectively, while the labeled values were 66, 70, 80, 70, 66%. The values are also tabulated in Table 2. The results indicate that the ethanol content of all the commercial hand sanitizers was lower than the labeled values. The results were compared to those obtained by GC and also found to be lower than the reported values (Table 2).

**Table 2:** Concentration of ethanol, isopropanol and methanol in various commercial hand sanitizers.

Sample	Type	Concentration (%)				
		Labeled	Measured			
			FTIR			GC
			C−O stretching (1000–1140 cm^−1^)	C−H stretching (2976 cm^−1^)	C−H bending (881 cm^−1^)	
A	Ethanol	66	59	33–34	42–43	40
B	Ethanol	70	63	62–63	60–61	69
C	Ethanol	80	62	78	70	65
D	Ethanol	70	35	79	75–76	45
E	Ethanol	66	45	57–58	56	44
				2970 cm^−1^	CH_3_−C−CH_3_ stretching (952 cm^−1^)	
F	IPA	65	–	56	58–59	61
G	IPA	70	–	64–65	64	61
H	IPA	70	–	57–58	60–61	66
			1032 cm^−1^	2943 cm^−1^		
I	Methanol	70	98	98	–	81
J	Methanol	Mentioned as alcohol	37–38	39	–	19

**Fig. 3:**
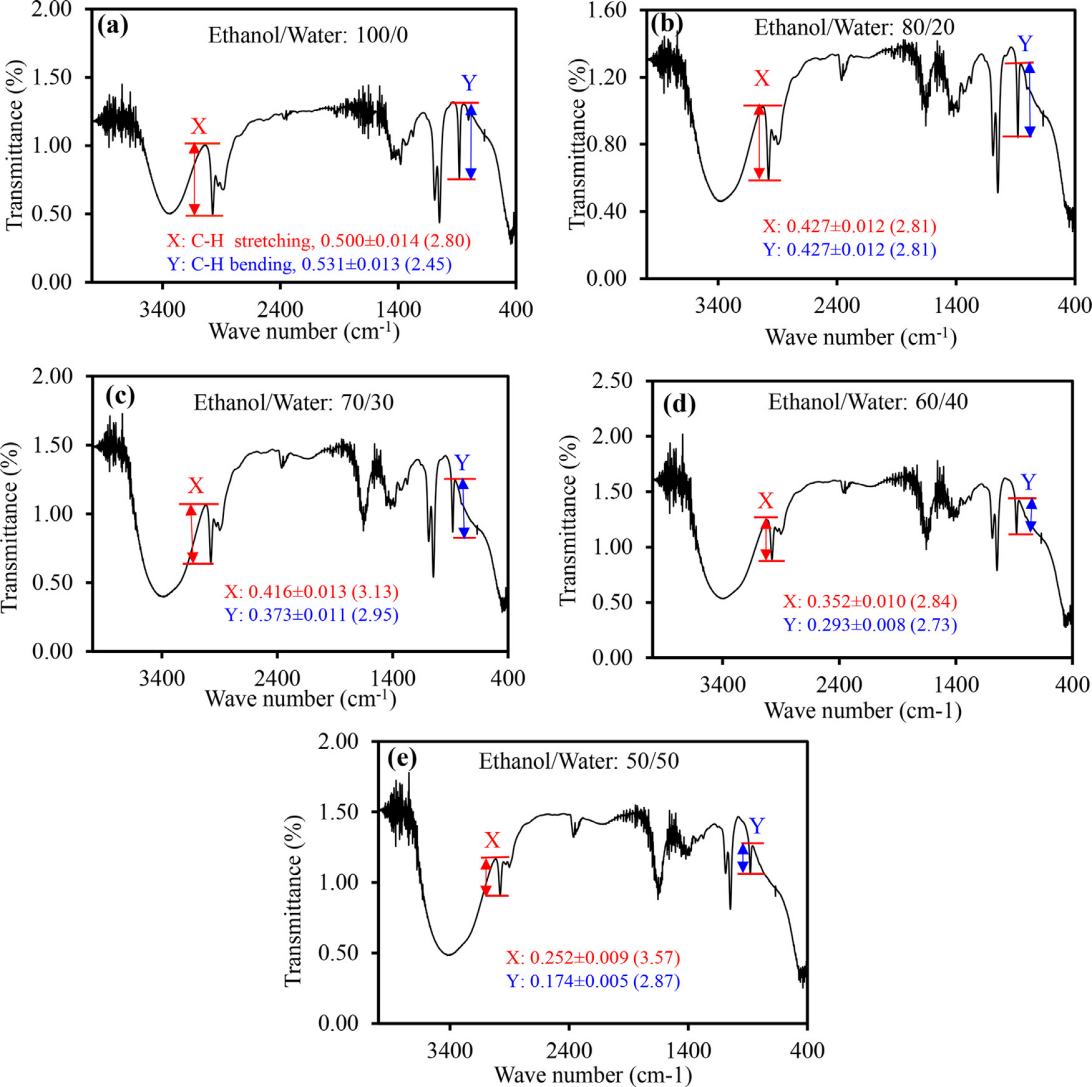
IR spectra of ethanol with different content of water (a) 0, (b) 20, (c) 30, (d) 40 and (e) 50%. The red and blue marks are for variation of heights of C−H stretching (∼2975 cm^−1^) and C−H bending (881 cm^−1^) vibrational modes with ethanol/water ratios, respectively. RSD are in the parentheses.

The IR spectra of ethanol with varying water content (a) 0, (b) 20, (c) 30, (d) 40, and (e) 50 percent are shown in Figs. 3(a)-(e). The red and blue marks indicate how ethanol/water ratios affect the intensities of C−H stretching (2975 cm^−1^) and C−H bending (881 cm-1) vibrational modes, respectively. The height of both peaks (C−H stretching and C−H bending) decreases with increasing water content, as illustrated in Figs. 3(a)-(e). Accordingly, two analytical calibration curves based on variation in heights of the C−H stretching and C−H bending vibration modes were constructed, as illustrated in Figures S4(a) and (b). For the two curves, the correlation coefficients (*R*^2^) were 0.905 for C-H stretching and 0.980 for C−H bending vibration mode. According to the findings, using the C−H bending vibration mode to quantify ethanol in hand sanitizers could be more precise.

**Fig. 4:**
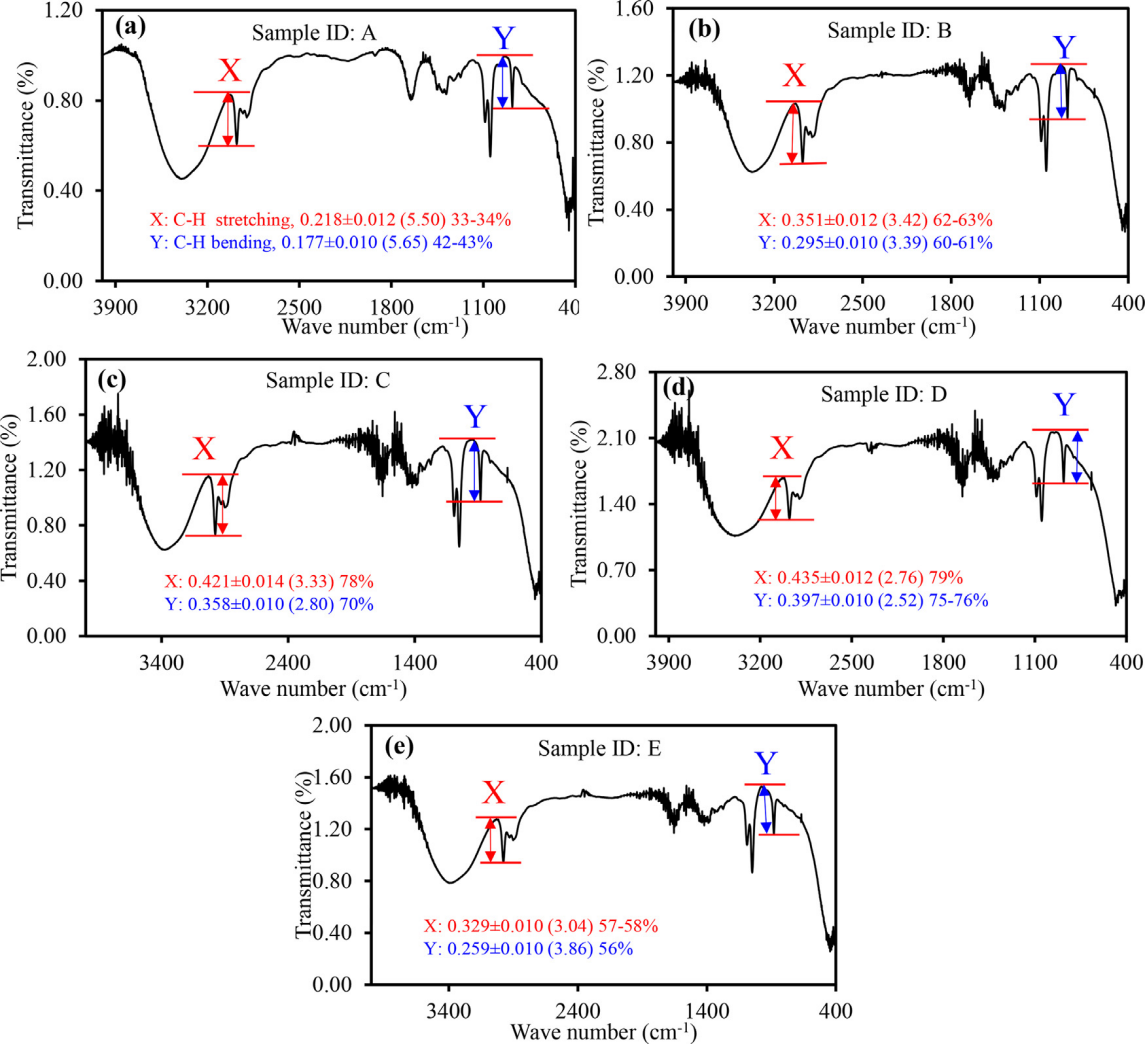
IR spectra of commercial hand sanitizers (a) A, (b) B, (c) C, (d) D and (e) F. The red and blue marks are for variation of heights of C−H stretching (∼2975 cm^−1^) and C−H bending (881 cm^−1^) vibrational modes with ethanol/water ratios, respectively. RSD are in the parentheses.

With the exception of sample D, the content of ethanol in commercial hand sanitizers was lower than the reported values, as revealed by the analytical curves in Figures S4(a) and (b). Table 2 and Figs. 4(a)-(e) show the results. The results were compared to those of GC. For samples A, B, and C, the ethanol content was comparable using these two methods, FTIR and GC, where the C−H bending vibration-based analytical curve exhibiting more precise results (Table 2). However, the ethanol content of samples D and E was much higher by both calibration curves (C−H stretching and C−H bending) than those examined by GC, but they were quite close to the labeled values. The ethanol content measured by the C−O stretching calibration curve was similar to that measured by GC, however it was significantly lower than the reported values (Table 2).

According to WHO formulations, hand sanitizers are supposed to contain ethanol (75–80%) or IPA (70–75%), as well as glycerol (1.45%), hydrogen peroxide (0.125%), and water. Because both water and glycerin molecules make hydrogen bonds with ethanol molecules through the C−O−H group of ethanol and H−O−H and/or C−O−H of glycerin, the C−O stretching mode of alcohol can be dramatically changed by adding polar compounds like water, glycerin, and so on. This is why the C−O stretching mode has been used to determine the amount of alcohol in hand sanitizers [18,25].

Polar compounds, such as water or glycerin, on the other hand, do not interact with the C−H group of alcoholic compounds and instead reduce the peak height when they are added. As a result, employing the analytical calibration curves derived by C−H bending and/or C−H stretching to quantify alcoholic substances such as ethanol, isopropanol, and/or methanol in hand sanitizers is scientifically plausible.

**Fig. 5:**
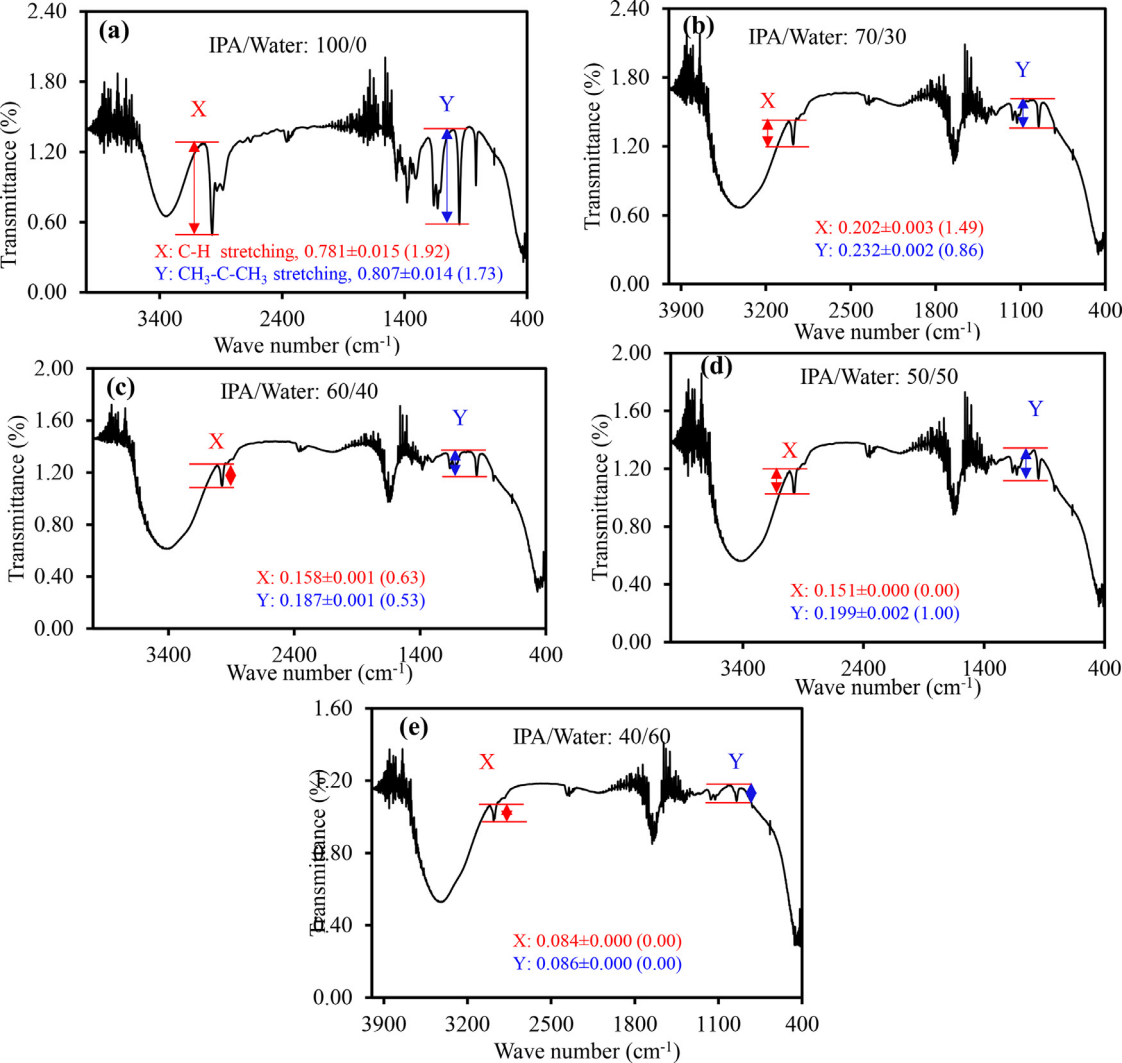
IR spectra of isopropanol (IPA) with different content of water (a) 0, (b) 30, (c) 40, (d) 50 and (e) 40%. The red and blue marks are for variation of heights of C−H stretching (∼2975 cm^−1^) and CH_3_−C−CH_3_ bending (952 cm^−1^) vibrational modes with IPA/water ratios, respectively. RSD are in the parentheses.

The IR spectra of isopropanol (IPA) with 0, 30, 40, 50, and 60 percent water are shown in Figs. 5(a)-(e), respectively. The spectral pattern of the IPA has substantially changed when water is added, as shown in Figs. 5(a)-(e). When water was added, the intensities of both the C−H and C−O stretching vibrational modes decreased dramatically, in contrast to ethanol. The bending vibration of the CH_3_−C−CH_3_ group caused an additional peak for IPA at 952 cm^−1^. With the addition of water, the intensity of this peak was also reduced. As shown in Figures S5(a) and (b), C−H (2970 cm^−1^) and CH_3_−C−CH_3_ (952 cm^−1^) bending were used to construct analytical calibration curves for detecting and quantifying IPA in commercial hand sanitizers (Table 1). In the calibration curves, the correlation coefficients (*R*^2^) for C−H and CH_3_−C−CH_3_ bending were found to be 0.913 and 0.922, respectively. This is due to the fact that the presence of polar water molecules has a considerable impact on the C−H and CH_3_−C−CH_3_ bending vibration modes of IPA, resulting in a regular fluctuation in peak height with the addition of water.

Like ethanol, an analytical calibration curve based on C−O stretching was also constructed for IPA and correlation coefficient (*R*^2^) was found to be less than 0.700 (data not shown). Density functional theory (DFT) calculation supported the deformation of IPA molecules in presence of water [26]. They reported that the aqua-IPA changes their structure from a planar ring structure to a three-dimensional cage structure when the number of water molecules around IPA become 5 (IPA-(H_2_O)_5_). Unlike ethanol, IPA molecules are less polar because of presence of two methyl group. So, intensities of the both C−H and CH_3_−C−CH_3_ bending are sensible in presence of water, accordingly two analytical curves were constructed for IPA.

The IR spectra of commercial hand sanitizers of F, G, and H are shown in Figure S8(a), (b), and (c), respectively. IPA content in hand sanitizers was estimated using the two analytical calibration curves (Figure S5(a) and (b)) and listed in Table 2. The results show that IPA content based on the CH_3_−C−CH_3_ peak is extremely similar to GC values. The C−H stretching-based calibration curve showed similar results to the GC values (Table 2). None of the IPA values in commercial hand sanitizers meet the FITR and/or GC-estimated values.

As expected, methanol is found in two out of ten hand sanitizers in Bangladesh. Alcohol dehydrogenase converts methanol into formaldehyde, which is then transformed to formic acid by aldehyde dehydrogenase. If ingested at a high enough quantity, it can result in blindness and death. The IR spectra of methanol with 0, 20, 30, and 40% water are shown in Figure S9(a)-(d), respectively. The red and blue marks indicate how methanol/water ratios affect the heights of C−H stretching (2975 cm^−1^) and C−O stretching (1032 cm^−1^) vibrational modes, respectively. Unlike IPA, methanol has a characteristic C−O stretching vibration mode that appears at 1032 cm^−1^ (Figure S9(a)) and subsequently drops in peak height when the amount of water increases (Figures S9(b)-(d)). The height of the C−H stretching (2943 cm^−1^) mode similarly lowers as the water content increases. In order to identify and quantify methanol in hand sanitizers, both the C−H and C−O stretching modes were used in the construction of two analytical calibration curves. Figures S6(a) and (b) illustrate the calibration curves based on C−H and C−O stretching vibrations, respectively. The correlation coefficients (*R*^2^) were found to be 0.904 and 0.996 for C−H and C−O stretching vibrations, respectively.

The IR spectra of commercial hand sanitizers I and J are shown in Figure S10(a) and (b), respectively. The red and blue marks represent the heights of the C−H stretching (2943 cm^−1^) and C−O stretching (1032 cm^−1^) vibrational modes, respectively, as a function of methanol/water ratios. Methanol content in I and J samples was 98 and 37–38% using the calibration curve-based on C−O stretching, respectively, while it was 98 and 39% using the analytical curve-based on C−H stretching, as shown in Figures S6(a) and (b) and Table 2. However, methanol content in samples I and J was found to be 81 and 19%, respectively, using GC (Table 2). According to manufacturers, sample I was written as 70% alcohol instead of methanol, whereas sample J was written as just alcoholic. The IR-based finding is less analytically valid if the methanol content in sample I determined by GC is correct. Adulteration with hydrocarbon-based compounds is offered as another analytical way to evaluate the quality of sample I, based on the high content detected by the IR-based method.

In order to evaluate the selectivity of the assigned wavenumbers for the identification of ethanol, IPA and methanol in the commercial hand sanitizers, the effect of additional alcohols and/or volatile organic compounds, such as acetone, 1-propanol, and ethyl acetate, that may be present in such formulations was assessed (data not shown). Since the concentrations of ethanol, IPA, and methanol in the samples are relatively high (30%–75%), no influence from other alcohols or volatile compounds like acetone, butanol, ethyl acetate, etc. occurs until their concentration level reaches the same order as the alcohols taken into consideration [27]. None of the samples used in the current study included mixed alcohols.

## CRediT authorship contribution statement

**Saima Alam:** Conceptualization, Data curation, Formal analysis, Investigation, Methodology. **Md. Masudur Rahman Rahat:** Data curation, Formal analysis, Writing – review & editing. **Nusrat Jahan Upoma:** Data curation, Formal analysis, Methodology. **Chandan Halder:** Formal analysis, Investigation, Writing – review & editing. **Shyama Prosad Moulick:** Methodology, Validation. **Md. Monarul Islam:** Methodology, Validation. **Wenben Liu:** Conceptualization, Writing – review & editing. **Ahsan Habib:** Conceptualization, Funding acquisition, Investigation, Validation, Visualization, Writing – original draft, Writing – review & editing.

## Declaration of Competing Interest

The authors declare that they have no known competing financial interests or personal relationships that could have appeared to influence the work reported in this paper.

## Data Availability

Data will be made available on request.

## Appendix Supplementary materials

Image, application 1
