# Supplementary material for: Assessment of quality of commercial hand sanitizers using Fourier transform infrared spectroscopy and gas chromatography
Source: MethodsX. 2023 Jul 6;11:102274. doi: 10.1016/j.mex.2023.102274 (PMC10362314; doi:10.1016/j.mex.2023.102274)
Supplement: Supplementary file 1 [file mmc1.docx]

**Assessment of quality of commercial hand sanitizers using Fourier transform infrared spectroscopy and gas chromatography**

Saima Alam^1^, Md. Masudur Rahman Rahat^1^, Nusrat Jahan Upoma^1^, Chandan Halder^1^, Shyama Prosad Moulick^2^, Md. Monarul Islam^2^, Wenben Liu^3^ and Ahsan Habib^1*^

^1^Department of Chemistry, University of Dhaka, Dhaka 1000, Bangladesh

^2^Bangladesh Council for Scientific and Industrial Research (BCSIR), Dhaka 1205, Bangladesh

3Research Center for Eco-Environmental Sciences, Chinese Academy of Sciences, Beijing 100085, China

-------------------------------------------------------------------

Corresponding author’s email: habibchem@du.ac.bd

Ahsan Habib, Department of Chemistry, University of Dhaka, Dhaka 1000, Bangladesh.

Email: habibchem@du.ac.bd; Tel.: +880-1779227863; Fax: +880-2-55167810

Supplementary Figure S1. Photos of collected commercial hand sanitizers with (a) ethanol, (b) IPA and (c) methanol.

Supplementary Figure S2. Sample introducing into the calcium fluoride crystal made plates for recording IR spectrum.

Supplementary Figure S3. Analytical calibration curve for ethanol based on absorbance for the peak appearing at 1050 cm^-1^ at various ethanol-water ratios.

Supplementary Figure S4. Analytical calibration curves for ethanol with different ethanol-water ratios of (a) C-H stretching (2976 cm^-1^) and (b) C−H bending (881 cm^-1^) vibration modes.

Supplementary Figure S5. Analytical calibration curves for IPA with different IPA-water ratios (a) C−H stretching (2970 cm^-1^) and (b) CH_3_−C−CH_3_ bending (952 cm^-1^).

Supplementary Figure S6. Analytical calibration curves for methanol with different methanol-water ratios of (a) C−H stretching (2943 cm^-1^) and (b) C−O stretching (1032 cm^-1^).

Supplementary Figure S7. IR spectra of (a) ethanol (100%), (b) isopropanol (100%) and (c) methanol (100%). The red and blue circles indicate C−H and C−O stretching, respectively. The purple circle shows C−H bending while green and orange circles stand for -C(CH_3_)_2_ stretching and C−C−O symmetric stretching, respectively.

Supplementary Figure S8. IR spectra of commercial hand sanitizers of (a) F, (b) G and (c) H. The red and blue marks are for variation of heights of C−H stretching (~2975 cm^-1^) and CH_3_−C−CH_3_ bending (952 cm^-1^) vibrational modes with IPA-water ratios, respectively. RSD are in the parentheses.

Supplementary Figure S9. IR spectra of methanol with different content of water (a) 0, (b) 20, (c) 30, (d) and 40%. The red and blue marks are for variation of heights of C−H stretching (~2975 cm^-1^) and C−O stretching (1032 cm^-1^) vibrational modes with methanol/water ratios, respectively. RSD are in the parentheses.

Supplementary Figure S10. IR spectra of commercial hand sanitizers (a) I and (b) J. The red and blue marks are for variation of heights of C−H stretching (~2943 cm^-1^) and C−O stretching (1032 cm^-1^) vibrational modes with methanol/water ratios, respectively. RSD are in the parentheses.

## Supplementary Table S1. Analytical procedure in detecting and quantifying alcohol contents in commercially available hand sanitizers by GC-FID:

Typical chromatographic conditions

Detector: FID

Detector temperature: 250 ºC

Flow rate of gas:

| Mobile phase/gas | Flow rate (mL/min) |
| --- | --- |
| H_2_ gas | 40.0 |
| He gas | 1.5 |
| N_2_ gas | 25.0 |
| Air/O_2_ | 300.0 |

Column: SCION-5 column, Stationary Phase -5%-phenyl-methylpolysiloxane

Internal diameter - (30m x 0.25mm) and film thickness - 0.25µm.

Column Oven Temperature:

| Rate (ºC/min) | Temperature (ºC) | Time (min) | Total (min) |
| --- | --- | --- | --- |
| Initial | 40 | 1.0 | 1.00 |
| 5.0 | 80 | 2.0 | 11.00 |
| 20.0 | 120 | 1.0 | 14.00 |
|  |  | Total time | 14.00 |

Injection volume: 0.01 µL

Injection port Temperature: 250 ºC

Mode of Injection: Split ratio (1:50)

Run Time: 14 min.
